# A multicentre, randomised intervention study of the Paediatric Early Warning Score: study protocol for a randomised controlled trial

**DOI:** 10.1186/s13063-017-2011-7

**Published:** 2017-06-08

**Authors:** Claus Sixtus Jensen, Hanne Aagaard, Hanne Vebert Olesen, Hans Kirkegaard

**Affiliations:** 1 Department of Clinical Medicine, Aarhus University, Palle Juul-Jensens Boulevard 82, 8200 Aarhus N, Denmark; 2 Department of Paediatrics and Adolescent Medicine, Herlev Gentofte Hospital, Herlev Ringvej 75, 2730 Herlev, Denmark; 3 Research Centre for Emergency Medicine, Aarhus University Hospital, Trøjborgvej 72-74, Bygning 20, 8200 Aarhus N, Denmark; 4 Department of Child and Adolescent Health, Aarhus University Hospital, Palle Juul-Jensens Boulevard 99, 8200 Aarhus N, Denmark

**Keywords:** Paediatric Early Warning Score, Randomised controlled trial, Paediatrics, Intensive care unit

## Abstract

**Background:**

Patients’ evolving critical illness can be predicted and prevented. However, failure to identify the signs of critical illness and subsequent lack of appropriate action for patients developing acute and critical illness remain a problem. Challenges in assessing whether a child is critically ill may be due to children’s often uncharacteristic symptoms of serious illness. Children may seem relatively unaffected until shortly before circulatory and respiratory failure and cardiac arrest.

The Bedside Paediatric Early Warning Score has been validated in a large multinational study and is used in two regions in Denmark. However, healthcare professionals experience difficulties in relation to measuring blood pressure and to the lack of assessment of children’s level of consciousness. In addition, is it noteworthy that in 23,288-hour studies, all seven items of the Bedside Paediatric Early Warning Score were recorded in only 5.1% of patients.

This trial aims to compare two Paediatric Early Warning Score (PEWS) models to identify the better model for identifying acutely and critically ill children. The hypothesis is that the Central Denmark Region PEWS model is superior to the Bedside PEWS in terms of reducing unplanned transfers to intensive care or transfers from regional hospitals to the university hospital among already hospitalised children.

**Methods/design:**

This is a multicentre, randomised, controlled clinical trial where children are allocated to one of two different PEWS models. The study involves all paediatric departments and one emergency department in the Central Denmark Region. The primary outcome is unplanned transfer to the paediatric intensive care unit or transfer from regional hospitals to the university hospital. Based on preliminary data, 14,000 children should be included to gain a power of 80% (with a 5% significance level) and to detect a clinically significant difference of 30% of unplanned transfers to intensive care or from regional hospitals to the paediatric department at the university department. A safety interim analysis will be performed after inclusion of 7000 patients.

**Discussion:**

This is the first randomised trial to investigate two different PEWS models. This study demonstrates the safety and effectiveness of a new PEWS model and contributes to knowledge of hospitalised children’s clinical deterioration.

**Trial registration:**

ClinicalTrials.gov, NCT02433327. Registered on 27 April 2015.

**Electronic supplementary material:**

The online version of this article (doi:10.1186/s13063-017-2011-7) contains supplementary material, which is available to authorized users.

## Background

Patients’ evolving critical illness and death can be predicted and prevented [1, 2]. The clinical deterioration of hospitalised patients is often preceded by physiological changes up to 24 hours before death [2, 3]. The care and treatment of critically ill children is a particular challenge, as children’s symptoms of critical illness can be uncharacteristic. A child with sepsis or severe dehydration can seem relatively unaffected, and the acute condition is often only identified by the affected vital parameters [4]. Children’s compensatory mechanisms are better than those of adults because they are able to maintain an almost normal blood pressure despite considerable loss of fluid. On the other hand, a child’s condition can deteriorate almost immediately when his/her ability to compensate is overwhelmed. Thus, it is important to identify and act on the often subtle signs of acute and critical illness in a child, as the prognosis for survival if a child develops cardiac arrest is very poor [5]. International studies have shown that between 8% and 14% of cardiac arrest incidences in intensive care units involve paediatric patients [6]. The rate of survival is only between 15% and 33%, with subsequent very poor neurological outcomes in 35% of those who survive [7]. A study of 126 deaths among English children showed that 89 deaths occurred in hospital; among them, 63 (71%) could have been avoided [8]. The difficulty of recognising the severity of the disease was a decisive factor, as was the inability to measure and interpret physical signs correctly. To provide timely care and treatment for children, systematic assessment of children’s symptoms and their severity is vital. If health professionals do not systematically observe, interpret and act adequately to address changes in a child’s condition, a hospitalised child is likely to experience serious consequences [8]. The Paediatric Early Warning Score (PEWS) is a system for identifying clinically deteriorating children. The PEWS tool is a simple physiological scoring system that can be applied at the patient’s bedside using parameters that can easily be measured without complex, expensive equipment.

The Canadian Bedside PEWS model has proven to be at least equally good as if not superior to other paediatric early warning system models, with a sensitivity of 83% and a specificity of 95% [9–11]. The model has been developed, tested and modified and now comprises seven different parameters. However, it includes the measurement of blood pressure, which is a specific challenge in the paediatric population. Children are often upset when their blood pressure is measured, which either causes the pressure to increase (not necessarily a sign of clinical exacerbation of disease) or results in failure to obtain blood pressure measurements. At the same time, low blood pressure is a late indicator in connection with evolving critical illness due to children’s ability to compensate; this could be an argument for not measuring the blood pressure of all hospitalised children [5]. Moreover, the model does not include the assessment of children’s level of consciousness, which is an important factor for assessing whether critical illness is evolving in children. Finally, note that all seven items were recorded in only 5.1% of patients in 23,288-hour studies [11].

In the present study, we compare the Canadian model with the Central Denmark Region model, which includes the assessment of the level of consciousness, but not the measurement of blood pressure. Blood pressure measurement in the Central Denmark Region model will be performed as an additional optional measurement in line with other additional measurements such as urine output. It could be relevant among children with a high PEWS value, i.e. children at risk of evolving critical illness or children whose illness requires blood pressure measurement. Blood pressure measurement will thus be a prescribed action and will not be used as a screening mechanism for all hospitalised children as in the Bedside PEWS model. Apart from this, the two PEWS models are similar in terms of their underlying action algorithms, age categories and cut-off values for vital signs.

This manuscript is prepared according to the Standard Protocol Items: Recommendations for Interventional Trials (SPIRIT) guidelines [12].

### Objective

The aim of this study was to compare the ability of the two PEWS models to reduce the number of unplanned admissions to intensive care or the number of transfers from regional hospitals to the university hospital among already hospitalised children due to clinical deterioration.

### Hypothesis

The hypothesis is that the Central Denmark Region PEWS is superior to the Bedside PEWS in terms of reducing hospitalised children’s unplanned transfers to the intensive care unit and transfers from regional hospitals to the university hospital due to clinical deterioration requiring proximity to a paediatric intensive care unit (PICU).

### Trial design

This is a multicentre, randomised, controlled clinical intervention study.

## Methods/design

### Study setting

The study is ongoing in four paediatric departments, four paediatric emergency and assessment units, one emergency department and a specialised paediatric unit for children with neurological illness in the Central Denmark Region. Including both a university hospital and several regional hospitals helps ensure the highest possible diversity in the population and strengthens the study’s generalisability. The participating departments are listed on the project homepage (www.pews.dk). None of the participating hospitals had been working with any kind of PEWS model prior to this study.

### Eligibility criteria

Participants are aged 18 years and below.

Inclusion criteria are as follows:

- All children admitted to the participating departments
- All children examined at the acute paediatric assessment units in the Central Denmark Region

Exclusion criteria are as follows:

- Children admitted directly to neonatal wards
- Children admitted directly to intensive care units
- Children dead upon arrival at the hospital
- Children admitted due to social interaction problems
- Children receiving palliative care
- Children whose informed consent was not obtained

### Interventions

Both of the PEWS tools in this project consist of seven parameters (Table 1). Each individual parameter generates a score of 0, 1, 2 or 4; these are accumulated to a total score. See Additional file 1 for a more detailed a description of the scoring systems.

**Table 1:** Contents of the two PEWS models

Bedside PEWS model	Central Denmark Region model
Heart rate	Heart rate
Respiratory rate	Respiratory rate
Respiratory effort	Respiratory effort
Systolic blood pressure	Level of consciousness
Pulse oximetry	Pulse oximetry
Oxygen therapy	Oxygen therapy
Capillary refill time	Capillary refill time

The two PEWS models investigated in the present study are incorporated into a management system that consists of the following elements:

1. An electronic patient chart with unique software that provides an age-specific sub-score for each of the seven clinical observations and an accumulated total PEWS value
2. An action algorithm providing a set of minimal actions to assist deteriorating children by involving all necessary team members in a timely manner; the algorithm also assists in determining the level of necessary care (see Table *2*)

**Table 2:** Decision algorithm for both PEWS models

PEWS value	Minimum observation interval	Decision algorithm
0	Every 12 hours	Continue scoring every 12 hours
1–2	Every 6 hours	Nursing staff ABCDE optimizes; see action card (shown in Additional file 2)
		If the score is 2, inform the nurse in charge and as a minimum, for one single score at 2, inform the nurse in charge before the physician
3–5	Every 4 hours	Nursing staff ABCDE optimizes; see action card
		If the score is 3 or above or as a minimum For one single score at 3, inform the physician. The physician is to make a plan of action
6	Every 2 hours	Nursing staff ABCDE optimizes; see action card
		Send for the physician
		The physician shall attend to the patient and make a plan of action. Any indication for taking blood pressure?
7–8	Every hour	Nursing staff ABCDE optimizes; see action card
		Send for the physician
		The physician shall attend to the patient within 30 minutes
		The physician confers with the specialist doctor
		The physician makes a plan of action. Any indication for taking blood pressure?
9 or above	Every 15 minutes	Nursing staff ABCDE optimizes; see action card
		The physician shall attend to the patient within 15 minutes
		The physician confers with the specialist doctor
		The physician makes a plan of action

*ABCDE* Airway, Breathing, Circulation, Disability, Exposure
3. Clinical guidelines
4. Educational programme for nurses and medical doctors

All nurses employed in the participating departments attended a 2-hour teaching session prior to implementing the PEWS system, while medical doctors attended a 30-minute teaching session. The children included in the study will be monitored using one of the two different PEWS models. Both models include the measurement of vital signs at different intervals according to the child’s condition. The PEWS will be measured on admission; action then follows as guided by the algorithm. If a child’s clinical condition is deteriorating, the ‘score’ for the observations will increase; a higher or increasing score gives an early indication that intervention may be required. Based on the results of the clinical assessment and PEWS value, nurses can find the corresponding action(s) on the PEWS action algorithm. The corresponding action could, for example, be more frequent reassessment or notifying the charge nurse or paediatrician.

Observations are documented in the electronic patient chart, which also provides information based on the PEWS value for observation intervals. Underlying both PEWS tools are algorithms of clinical decision support for the critically ill child based on the Airway, Breathing, Circulation, Disability, Exposure (ABCDE) principle (see Additional file 2), clinical guidelines and guidance for standardised monitoring.

### Outcomes

The primary outcome is the sum of unplanned transfers to the PICU and transfers from regional hospitals to the university hospital or the number of deaths. Transfers from regional hospitals to the paediatric department at the university hospital are equated with transfers to the PICU; children who need intensive care at the regional hospitals will be transferred to the paediatric department at the university hospital to ensure proximity to the PICU.

The secondary outcomes are as follows:

- Paediatric Index of Mortality score
- Severity of illness during PICU stay based on invasive ventilation and inotropes
- Length of hospital stay
- Length of PICU stay

#### Paediatric Index of Mortality

The Paediatric Index of Mortality (PIM3) is used to provide an estimate of mortality risk among children admitted to paediatric intensive care [13]. The PIM3 consists of 10 variables that should be collected within one hour after arrival at the PICU. The PIM3 score consists of physiological variables and categorical variables that classify patients based on the reason for admission, the use of mechanical ventilation and diagnostic risk data [13]. It is not meant to be used as a marker for the severity of illness of individual patients; however, like other mortality prediction models, it has been used to evaluate the risk of mortality when registering patients in clinical trials and as a tool for monitoring the quality of intensive care [13]. Thus, we also use invasive ventilation and inotropes as markers for the severity of illness.

### Sample size

A power calculation was made based on accessible data from 2013 from the departments that are currently employing PEWS models. The available preliminary data showed 154 unplanned transfers to the PICU out of 10,000 admissions. With a power of 80%, a 5% significance level and an expected 30% reduction in the number of transfers to intensive care, we would have to include 7112 children in each group. The total available study population is 26,800 children annually.

### Recruitment

**Fig. 1:**
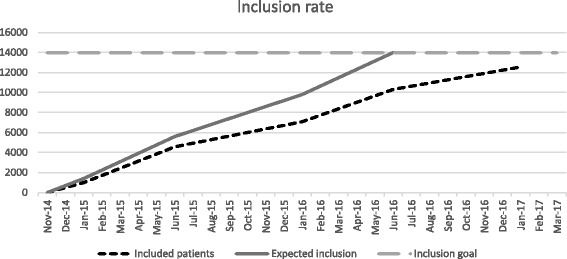
Inclusion rate

Recruitment of patients started on 12 November 2014 and is ongoing. As of January 2017, 12,000 patients have been randomised into one of the two arms. Inclusion of the final 2000 patients is expected to be reached by the end of March 2017 (Fig. 1).

### Allocation

The departments will all have access to a web-based randomisation programme, Trialpartner. Each patient receives his or her own randomised number. If a patient is readmitted during the inclusion period, observation will continue based on the result of the initial randomisation. Consecutive eligible patients are randomly allocated with a 1:1 ratio to one of the two PEWS tools. There is no stratification at the patient or institution level.

### Sequence generation

A data manager who is not involved in recruiting patients devised the randomisation procedure. Healthcare professionals in the participating departments will enrol and assign patients to the randomised PEWS model in the electronic patient chart. Patients, clinicians and the research team will not be blinded.

### Data collection and management

**Fig. 2:**
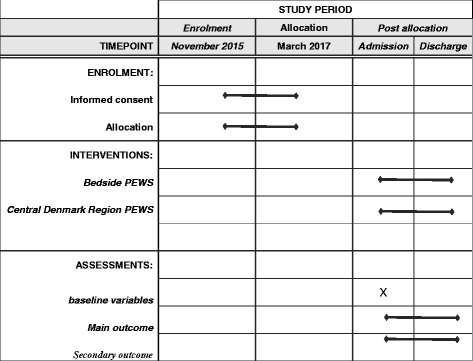
SPIRIT study schedule

The overall SPIRIT study schedule is illustrated in Fig. 2.

Separate interactive registration modules in the electronic patient chart were designed for each of the two PEWS models. The study data are extracted from the electronic patient charts. Thus, healthcare professionals in each department are responsible for data collection and data entry into the electronic patient chart as well as for obtaining consent from the parents.

For each patient included in the study, the following data are collected:

1. Baseline demographics: child’s age, gender, pre-existing conditions, diagnostic category (cardiac, post-surgical, neurological, respiratory, infection, hydration)
2. Clinical characteristics: discharge diagnosis, triage level at admission, time of day of critical event (day, evening, night)
3. PEWS parameters and temperature in the 24 hours preceding transfer to the PICU, ending one hour before transfer to the PICU
4. Frequency of PEWS and temperature measurements during the last 24 hours prior to transfer to the PICU, ending one hour before transfer to the PICU
5. Data from PICU: PIM3 score
6. Outcome data: unplanned transfer to the PICU or university hospital, length of hospital stay, length of PICU stay

Transfers to the PICU and transfers from regional hospitals to the university hospital will be identified from the electronic patient charts. Two researchers will review each of these medical records to exclude planned transfers. These are transfers that are elective or planned in advance, transfers coming directly from the operating room and transfers in which the need for PICU care was due to the need to recover from sedation.

This trial will follow the recommendations from the Consolidated Standards of Reporting Trials (CONSORT) statement for non-pharmacological treatment interventions.

### Statistical methods

Statistical analysis will follow the modified intention-to-treat principle [14]. Analysing the primary outcome, logistic regression of PEWS on unplanned PICU admissions will be performed to determine odds ratios (ORs) and 95% confidence intervals (CIs). The analysis will be adjusted for age. In the secondary outcome analysis, categorical data will be analysed using the chi-square test. Continuous data that are not normally distributed will be analysed using the Mann-Whitney *U* test. A *p* value <0.05 is considered significant.The two PEWS tools will also be evaluated using sensitivity, specificity and the receiver operating characteristic curve. In this study, sensitivity is the ability of the PEWS tool to correctly identify children at risk of an unplanned transfer to the PICU. Specificity is the ability of the tool to demonstrate a low score among children who are not at risk of an unplanned transfer to the PICU; thus, a low specificity indicates a high rate of false positives. Sensitivity and specificity will be calculated to measure the validity of the tools and the most predictive score for an unplanned transfer to the PICU. As the PEWS must be able to identify children at risk of acute life-threatening conditions and provide a ‘window’ for intervention, data analysis will stop one hour prior to the occurrence of the acute life-threatening condition to avoid overestimating the effect of the PEWS.

Descriptive statistics will be calculated to describe baseline variables and clinical characteristics. The descriptive analysis will be summarised as percentages, with median and interquartile ranges provided for continuous variables. STATA version 10 will be used for the analyses.

### Data monitoring

A Data and Safety Monitoring Committee (DSMC) will perform a safety interim analysis when 7000 of the 14,000 patients have been randomised. In the safety analysis, the two PEWS models will be compared in terms of transfers to the PICU or transfers from regional hospitals to the university hospital. An independent statistician will perform the analysis and provide the results to the DSMC. To give recommendations on whether the trial should terminate or continue, the DSMC will use the Haybittle-Peto statistical approach as a guide [15]. The final decision regarding termination or continuation of the trial will be made by the research group, which consists of the principal investigator and three other members. Depending on the recommendations of the DSMC, further safety analyses could be performed. The safety analysis has been performed, and the DSMC had no comments.

### Ethics and dissemination

The interventions directly affecting hospitalised children will be the measurement of pulse, saturation and blood pressure. The measurements are non-invasive; they cause no pain and only slight discomfort (blood pressure) and are standard procedures for most acutely ill, hospitalised children. Thus, they are expected to raise no concerns among children and parents. It is important that patients’ experiences of anxiety, concern or irritation are addressed. It is therefore crucial for ward nursing staff to inform patients about the rationale of the PEWS while they are measuring vital parameters and for nursing staff to be able to explain in a professional and clear manner why the measurement is important. Several studies have shown that bedside observations of vital parameters and simple algorithms based on these observations can identify patients at risk of deteriorating [9, 11, 16, 17]. It is therefore unethical to neglect reacting to these findings and not to implement a new ward practice.

### Dissemination policy

The results, whether positive, negative or inconclusive, will be published in a peer-reviewed international journal.

## Discussion

National and international models for early warning systems in adults exist [2, 18]. Nevertheless, several international reports have shown that failure to identify the signs of critical illness and subsequent lack of appropriate action for patients developing acute and critical illness remain a problem [19, 20].

In a PEWS review, Chapman et al. [21] concluded that there is considerable variation in the purpose, contents and threshold of actions of published PEWS models as well as a lack of evidence of their validity, reliability and applicability. Reliability was investigated only in one study, and no studies reported on health professionals’ experiences of the applicability of PEWS. In a British study of PEWS, there was a large variation in the use of parameters; 46 different parameters were used, and more hospitals used PEWS models that were not validated or thoroughly investigated [22]. A Cochrane review found that this also applies to early warning systems for adults, and the review drew attention to the lack of randomised controlled trials (RCTs) [2]. The present RCT compares two different PEWS tools.

Several different indicators of deterioration were considered as outcome measures for this trial. Cardiopulmonary arrest was initially considered, but other studies [9, 10, 23, 24] found it difficult to demonstrate an effect because the event rate of in-hospital cardiac arrest or death was low in this age group. Other proxies for the measurement of clinical deterioration were explored. Code-blue events were also considered as an indicator for identifying children at risk of clinical deterioration, but Duncan et al. [9] have discussed the limited number of code-blue events in relation to the challenge of developing screening mechanisms to identify children at risk of deterioration. Unplanned transfer to an intensive care unit has been used as a proxy for the measurement of clinical deterioration. Tucker et al. [25] demonstrated a statistically significant association between the PEWS and transfer to the PICU. In a retrospective case control study, Zhai et al. [26] used transfers to the PICU to develop an electronic health record-based automated algorithm and compared its effectiveness with that of two PEWS tools. For the present study, transfer to the PICU was chosen as a surrogate marker for clinical deterioration.

In conclusion, the development and implementation of the PEWS may prevent acute decompensation and the need for a higher level of care. It will help with early recognition and intervention on children at risk of deteriorating. The PEWS is also expected to assist in increasing healthcare professionals’ skills and competencies. This study will add to the limited body of knowledge of PEWS systems. It is also expected to contribute to deriving a common PEWS model in Denmark. Finally, a PEWS model can help reduce healthcare costs for society, as an intensive care hospital bed is more expensive than a hospital bed at a general paediatric department.

### Trial status

Patient inclusion in the PEWS project began in November 2014. All departments have been enrolling patients since March 2015. As of September 2016, 11,000 patients have been randomised. Patient recruitment is expected to end in February 2017.

## Additional files

Additional file 1: Description of the scoring systems: a detailed description of the two PEWS models. (PDF 482 kb)

Additional file 2: Clinical decision support: a detailed description of the clinical decision support action card. (PDF 463 kb)

Additional file 3: List of the participating departments. (PDF 202 kb)

Additional file 4: SPIRIT 2013 checklist: recommended items to address in a clinical trial protocol and related documents. (DOC 122 kb)

## Abbreviations

DSMC: Data and Safety Monitoring Committee
PEWS: Paediatric Early Warning Score
PICU: Paediatric intensive care unit
PIM3: Paediatric Index of Mortality
RCT: Randomised controlled trial
