# Supplementary material for: A multicentre, randomised intervention study of the Paediatric Early Warning Score: study protocol for a randomised controlled trial
Source: Trials. 2017 Jun 8;18:267. doi: 10.1186/s13063-017-2011-7 (PMC5465452; doi:10.1186/s13063-017-2011-7)
Supplement: Supplementary file 1 — Description of the scoring systems: a detailed description of the two PEWS models. (PDF 482 kb) [file 13063_2017_2011_MOESM1_ESM.pdf]

Central Denmark Region PEWS model

	Age group	0 point	1 point	2 point	4 point
Oxygen Therapy		Room air		<4 L./minute or <50%	> 4 L./minute or> 50%
Respiratory Effort		Normal	Mild increase	Moderate increase	Severe increase/any apnoea
Pulse Oximetry		> 94	91 - 94	< 90	
Respiratory Rate	0 til < 3 months	30-60	20-29 or 61-80	16-19 or 81-90	91-150 or 5-15
	3 til < 12 months	25-50	20-24 or 51-70	16-19 or 71-80	81-150 or 5-15
	1-4 years	20-40	16-19 or 41-60	13-15 or 61-70	71-150 or 5-12
	>4 - 12 years	20-30	15-19 or 31-40	11-14 or 41-50	51-150 or 5-10
	> 12 years	12-16	11 or 17-22	10 or 23-29	30-150 or 5-9
Heart Rate	0 til < 3 months	110-150	91-109 or 151-179	81-90 or 180-189	190-350 or 20-80
	3 til < 12 months	100-150	99-81 or 151-169	71-80 or 170-179	180-350 or 20-70
	1-4 years	90-120	71-89 or 121-149	61-70 or 150-169	170-350 or 20-60
	>4 - 12 years	70-110	61-69 or 111-129	51-60 or 130-149	150-350 or 20-50
	> 12 years	60-100	51-59 or 101-119	41-50 or 120-139	140-350 or 20-40
Capillary Refil Time		<3 seconds			≥ 3 seconds
Level of consciousness		Alert	Voice responsive	Pain responsive	Unresponsive

Bedside PEWS model

	Age group	0 point	1 point	2 point	4 point
Oxygen Therapy		Room air		<4 L./minute or <50%	> 4 L./minute or> 50%
Respiratory Effort		Normal	Mild increase	Moderate increase	Severe increase/any apnoea
Pulse Oximetry		> 94	91 - 94	< 90	
Respiratory Rate	0 to < 3 months	30-60	20-29 or 61-80	16-19 or 81-90	91-150 or 5-15
	3 to <12 months	25-50	20-24 or 51-70	16-19 or 71-80	81-150 or 5-15
	1-4 years	20-40	16-19 or 41-60	13-15 or 61-70	71-150 or 5-12
	>4 - 12 years	20-30	15-19 or 31-40	11-14 or 41-50	51-150 or 5-10
	> 12 years	12-16	11 or 17-22	10 or 23-29	30-150 or 5-9
Systolic blood pressure	0 to <3 months	60-80	51-59 or 81-99	46-50 or 100-129	130-200 or 30-45
	3 to <12 months	80-99	71-79 or 100-119	61-70 or 120-149	150-200 or 30-60
	1-4 years	90-110	76-89 or 111-124	66-75 or 125-159	160-200 or 30-65
	>4 - 12 years	90-120	81-89 or 121-139	71-80 or 140-169	170-250 or 30-70
	> 12 years	100-13	86-99 or 131-149	76-85 or 150-189	190-300 or 30-75
Heart Rate	0 to <3 months	110-150	91-109 or 151-179	81-90 or 180-189	190-350 or 20-80
	3 to <12 months	100-150	99-81 or 151-169	71-80 or 170-179	180-350 or 20-70
	1-4 years	90-120	71-89 or 121-149	61-70 or 150-169	170-350 or 20-60
	>4 - 12 years	70-110	61-69 or 111-129	51-60 or 130-149	150-350 or 20-50
	> 12 years	60-100	51-59 or 101-119	41-50 or 120-139	140-350 or 20-40
Capillary Refil Time		<3 seconds			≥ 3 seconds
