# Supplementary material for: A multicentre, randomised intervention study of the Paediatric Early Warning Score: study protocol for a randomised controlled trial
Source: Trials. 2017 Jun 8;18:267. doi: 10.1186/s13063-017-2011-7 (PMC5465452; doi:10.1186/s13063-017-2011-7)
Supplement: Supplementary file 2 — Clinical decision support: a detailed description of the clinical decision support action card. (PDF 463 kb) [file 13063_2017_2011_MOESM2_ESM.pdf]

CLINICAL DECISION SUPPORT		SEE IT, BECAUSE YOU ARE LOOKING FOR IT!	
**LOOK – LISTEN - FEEL**	**Assess the patient – symptoms?**	**Possible causes**	**Suggestions for action -** Assess the need for help!
**A Airways**	**Patent?**<br>• Free respiration without stridor, hoarseness, snoring or rattling secretions<br>**At risk?**<br>• The following may occur: stridor, hoarseness, snoring or rattling secretions<br>**Obstructed?**<br>• No or very little air exchange<br>• Movement of the thorax does not mean that the airways are free	• Reduced level of consciousness (tongue falls back)<br>• Foreign objects<br>• Swelling of the mouth, throat, neck	• Open airways: positioning of the head and jaw lift<br>• Provide oxygen until saturation exceeds 95% (mask with reservoir or nasal oxygen catheter)<br>• Suction in case of secretion issues<br>• Change in position?<br>• Remove any visible foreign bodies<br>• Consider adrenaline inhalation in case of stridor
**B Respiration**	**Saturation <94**<br>• Is the respiratory rate normal for the child's age?<br>• What type of respiratory movements (shallow/deep)<br>• How is the respiration (inhalation, use of auxiliary muscles, prolonged expiration, movement of the nostrils)<br>See the respiration symptom card<br>• How is the depth of the respiration - Tidal volume<br>-	• Asthma<br>• RSV or other viral airway infection<br>• Pneumonia<br>• Sepsis<br>• Fever<br>• Reduced level of consciousness<br>• Pneumothorax, pulmonary congestion, pulmonary oedema	• Provide oxygen until saturation exceeds 95% (mask with reservoir or nasal oxygen catheter)<br>• Change in position?<br>• Inhalation with, e.g., beta2-agonist<br>• Mask ventilation if:<br>- saturation decreases despite oxygen provision<br>- respiration is failing<br>• Consider inserting a ventricular probe<br>• Consider the acid-base status<br>***Re-assess A-B go to C***
**C Circulation**	**Is the pulse normal for the child's age?**<br>• How is central and peripheral pulse quality<br>• Is the pulse irregular<br>• How does the skin look (pale, cyanotic, flushed)<br>• What is the central capillary refill time (normal ≤ 2 sec)<br>• What is the blood pressure<br>• Signs of hepatic enlargement	• Dehydration<br>• Infection<br>• Heart issues<br>• Haemorrhage<br>• Sepsis<br>• Anaphylaxis/medicine-/blood reaction	• Provide oxygen until the saturation exceeds 95% (mask with reservoir or nasal oxygen catheter)<br>• Acid-base status with lactate<br>• Trendelenburg<br>• IV access + liquid bolus<br>• IO access + liquid bolus<br>• Blood transfusion (in case of haemorrhage)<br>• Cardiac arrest-equipment<br>• ECG<br>• Weight

			• Prostaglandin on suspicion of ductal-dependent heart condition<br>• Is there a difference between the saturation or blood pressure in both arms and legs<br><br>***Re-assess A-B-C go to D***
**D Level of consciousness**	**Normal level of consciousness?**<br>• Observe the level of consciousness (AVPU; Alert, responds to Voice, Pain or Unresponsive)<br>• Pupils (same/different, small/large)<br>• Tonus<br>• Stiffness of the neck and back<br>• Blood glucose<br>• Sudden change in consciousness/behaviour<br>• Changed pattern of movement (same/different for each side)<br>• Headache<br>• Convulsions	• O2 deficiency<br>• High CO2<br>• Low BP<br>• High/low BG<br>• Electrolyte disturbances<br>• Intoxication (medication, alcohol)<br>• Head trauma	• Provide oxygen until saturation exceeds 95% (mask with reservoir or nasal oxygen catheter)<br>• Target BS<br>• Glucose 10% in case of low blood sugar<br>• In case of reduced consciousness corresponding to V on the AVPU perform full GCS test<br>• In case of reduced consciousness corresponding to P on the AVPU or GCS < 9, consider ventilation by mask and intubation<br>• Consider antidote<br>• Neurological assessment<br>• Other examination such as:<br>lumbar puncture, CT or blood samples<br><br>***Re-assess A-B-C-D go to E***
**E Exposure**	**Temperature**<br>• Are there factors that affect the patient?<br>Lift the covers and look at the whole patient.<br>• Infection count?<br>• Electrolytes<br>• Blood glucose<br>• Lactate	• Infection/sepsis<br>• Cooling<br>• Trauma<br>• Lactate, base excess and pH are affected by lack of oxygen at the cellular level	• Search for infectious focus<br>• Antibiotics<br>• Expose/undress the patient<br><br>***Re-assess A-B-C-D-E***
