# Supplementary material for: A multicentre, randomised intervention study of the Paediatric Early Warning Score: study protocol for a randomised controlled trial
Source: Trials. 2017 Jun 8;18:267. doi: 10.1186/s13063-017-2011-7 (PMC5465452; doi:10.1186/s13063-017-2011-7)
Supplement: Supplementary file 3 — List of the participating departments. (PDF 202 kb) [file 13063_2017_2011_MOESM3_ESM.pdf]

The participating departments

Aarhus University Hospital.

- Department of Paediatrics and Adolescents Health
- Department of children's Orthopaedics

Herning Regional Hospital:

- Department of Paediatrics
- Emergency Department

Randers Regional Hospital:

- Department of Paediatrics

Viborg Regional Hospital:

- Department of paediatrics
